# Relationship between Ulcerative Colitis and Rheumatoid Arthritis: A Review

**DOI:** 10.7759/cureus.5695

**Published:** 2019-09-18

**Authors:** Mark G Attalla, Sangeeta B Singh, Raheela Khalid, Musab Umair, Emmanuel Epenge

**Affiliations:** 1 Research, California Institute of Behavioral Neurosciences and Psychology, Fairfield, USA; 2 Urology, California Institute of Behavioral Neurosciences and Psychology, Fairfield, USA; 3 Neurology, California Institute of Behavioral Neurosciences and Psychology, Fairfield, USA

**Keywords:** rheumatoid arthritis, ulcerative colitis, colitic arthritis

## Abstract

Ulcerative colitis (UC) is a colonic disease characterized by chronic inflammation. Rheumatoid arthritis (RA) is a rheumatological chronic inflammatory disease characterized by joint swelling and tenderness. It is also considered an autoimmune disorder. We want to discover if a link exists between UC and RA and if so, how UC affects the progress of arthritis.

We used PRISMA guidelines. In this study, we used PubMed, PubMed Central (PMC), and Google Scholar to collect data. Studies conducted more than 50 years ago, non-English articles, and animal studies were excluded. All types of studies were included. We used keywords like "ulcerative colitis", "rheumatoid arthritis", or "colitic arthritis" in the search.

We identified the following sets of results: 187,611 PubMed studies, 197,610 PMC studies, and 2,282,000 Google scholar studies. After applying inclusion and exclusion criteria, the number of appropriate studies was narrowed down to 50.

Arthritis is the most common complication of ulcerative UC. The radiological changes are similar to those seen in RA. There are common genes and antigens found in both diseases, such as human leukocyte antigen (HLA-B27), interleukin 15, IgA. Certain drugs used for the treatment of both disorders, including omega-3. Many studies revealed that a large number of patients with UC developed RA within a few years.

All the findings prove that there is a relation between ulcerative colitis and rheumatoid arthritis. This study is useful for doctors, scientists, and patients.

## Introduction and background

“As with many life-altering events, an autoimmune illness is almost guaranteed to cause you to re-evaluate your priorities.” ⁠-Joan Friedlander

Ulcerative colitis (UC) is an immune disorder of the colon characterized by chronic inflammation. The cause of the immune response is unclear, but genetic, dietary, and environmental risk factors all play a role [1].

In contrast with that of Crohn's disease (CD), the inflammation of ulcerative colitis is limited to the mucosa of the colon. The annual incidence of UC in the United States (US) is between nine and twelve cases per 100,000 persons [1]. Inflammatory bowel diseases are more common in industrialized countries and Western nations. The incidence levels also increased in persons who live at higher latitudes. Smokers and patients who have had an appendectomy are less likely to develop ulcerative colitis. The incidence of UC is equal between women and men, in contrast to CD, which has a higher incidence in women. UC often presents with abdominal pain, hematochezia, and diarrhea [1]. The onset of these symptoms may occur suddenly or gradually. About one-third of patients with UC have extraintestinal manifestations, with the most common one being arthritis (21% )[1].

Rheumatoid arthritis (RA) is a rheumatological chronic inflammatory disease characterized by joint swelling, joint tenderness, and the destruction of these synovial joints, leading to severe disability [2]. It is considered an autoimmune disease. RA is regarded as the most commonly diagnosed form of arthritis: inflammatory arthritis. The etiology of RA is multifactorial. Genetic susceptibility is evident in monozygotic twin and familial clustering studies, with a 50 percent risk of RA attributable to genetic factors. A study in the UK found that the population minimum prevalence of RA is 0.44% in men and 1.16% in women [2]. Patients with RA typically present with stiffness and pain in multiple joints. Respectively the wrists, proximal interphalangeal joints, and metacarpophalangeal joints are most commonly involved, and if the morning stiffness lasts more than one hour, it suggests an inflammatory etiology. It may present with boggy swelling due to synovitis [2]. Patients may also present with more indolent arthralgia before the onset of clinically apparent joint swelling. Systemic symptoms of weight loss, fatigue, and low-grade fever may occur with active disease [2].

Both UC and RA involve an immune response that is inappropriate or excessive. These autoimmune diseases can be caused or accompanied by a systemic disruption that may result in acute or chronic injury, sometimes severe, in any organ system. They have common inflammatory pathways; patients with one condition have a higher risk of having another of these diseases relative to the rest of the population. Individually, these autoimmune diseases are rare.

However, is there a relation or link between UC and RA? How can a patient with UC subsequently develop RA? How does ulcerative colitis affect the progress, prevalence, symptoms, and signs of RA? The answer to all these questions will create a clearer and fuller picture of UC and RA. It will improve the treatment of these cases, limit the appearance of arthritis in the colitic patient, and prevent the complications associated with these diseases.

This study aims to discover the link between these two diseases and how ulcerative colitis affects the progress of RA. We used the US National Library of Medicine (PubMed), PubMed Central (PMC), and Google Scholar to access appropriate data. This review presents the assimilated information from several articles, reviews, case reports, case studies, cohort studies, and clinical trials.

## Review

Methods

Literature Search

We followed the PRISMA guidelines for data collection and presentation in this study.

Search Strategy and Study Selection

The search strategy was designed and executed using data obtained from PubMed, PMC, and Google Scholar to identify the link between UC and RA. The search query employed both a list of keywords and index terminology including: "ulcerative colitis", “rheumatoid arthritis” and “colitic arthritis." We excluded animal studies and articles published more than 50 years ago. We included all types of studies published in English, including systemic reviews, clinical trials, case reports, and traditional reviews. In the study, we did not specify a particular country but included all countries worldwide. We included all full-text studies and abstracts with information about the link between UC and RA.

Role of the Funding Source

The funder of the search had no role in the study design, data collection, data interpretation, data analysis, or writing any of the reports. All the studies and the data of the search were collected legally. The author had full access to all data in the study; the corresponding author had the final responsibilities for the decision to submit for publication. 

Results

**Figure 1:**
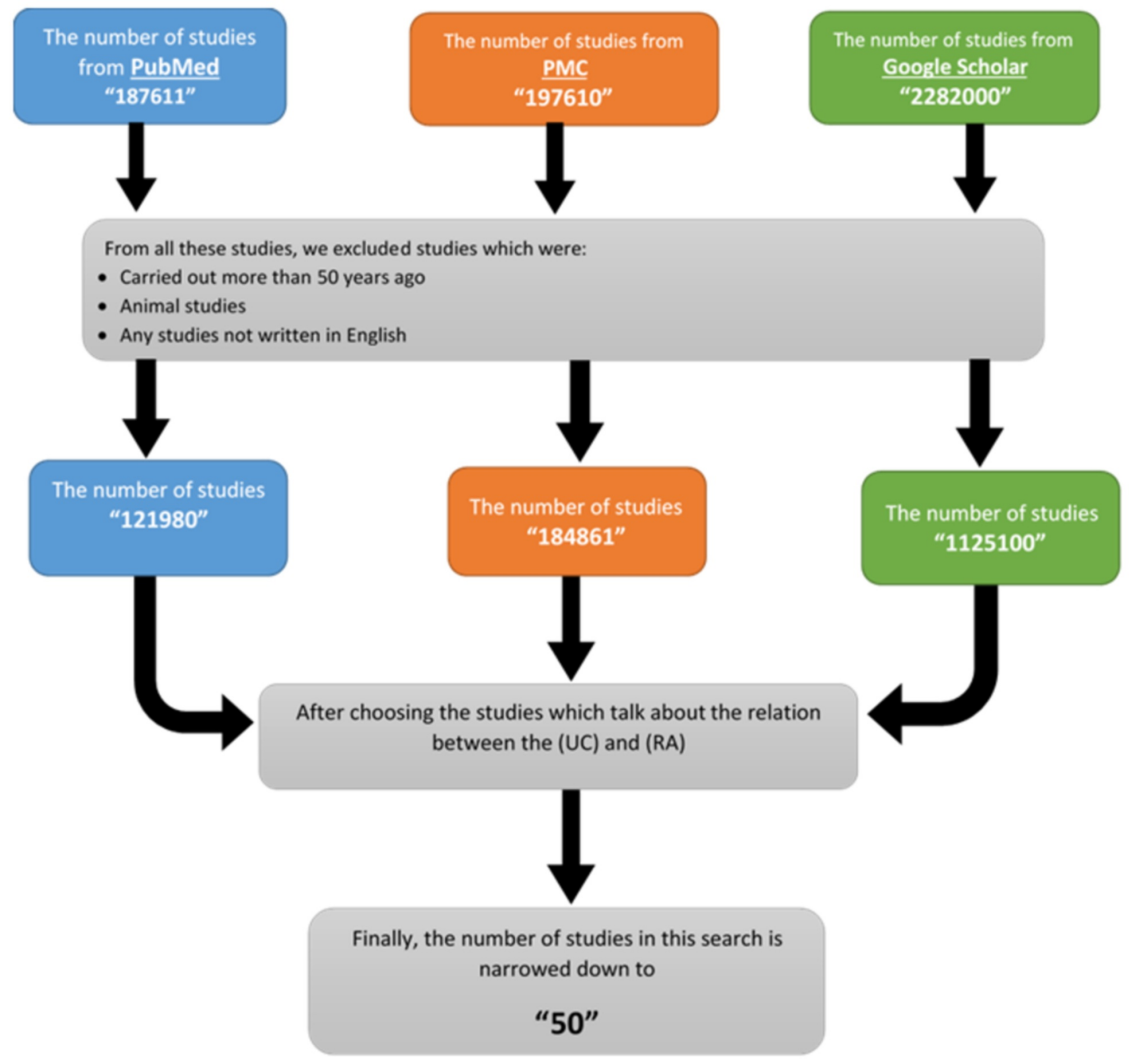
Flowchart describing the search strategy with the inclusion and exclusion criteria

The results for our methods and the inclusion and exclusion criteria are shown in Figure 1.

Discussion

UC and RA involve an immune response. These autoimmune diseases can be caused, presented, or accompanied by the systemic disruption that may result in acute or chronic inflammatory injury. However, is there a relation or link between UC and RA? How does RA develop in a patient with UC? We will discuss how UC affects the progress, the prevalence, the symptoms, and signs of RA.

The Characteristics of Arthritis in Ulcerative Colitis

Arthritis is the most common complication of UC [3,4]. The rheumatologic complications of UC may produce higher morbidity than the underlying intestinal disease [5]. Three studies describe the characteristics of arthritis [6-8]. Two studies characterized it as recurrent, earlier onset, and with acute asymmetrical involvement; usually monoarticular, the attacks were of short duration and accompanied with little residual deformity or disability [6,7]. The study by Wright and Watkinson is better as it contains several patients and has more references [6]. The third study suggested that peripheral arthritis might change from acute and nondestructive to chronic and destructive in some cases [8]. Sometimes there is an effusion in the affected joints, but the aspiration is sterile [3]. However, the serological test for the rheumatoid factor, which is always positive in RA, is negative [4,9].

Gravallese and Kantrowitz divided the rheumatological conditions associated with inflammatory bowel disease into four categories [10]. First, a unique form of peripheral arthritis which occurs in 15-20% of patients and is the self-limited, nondeforming, and seronegative type that waxes and wanes with bowel flares. Second, spondylitis which occurs in 3-6% of patients and is clinically and radiographically similar to idiopathic ankylosing spondylitis. Third, a bilateral, symmetrical sacroiliitis which occurs in 4-18% of patients. Fourth, a category that includes the rheumatologic complications of inflammatory bowel disease like clubbing, vasculitis, osteoporosis, and septic arthritis [10].

Also, the radiological changes are similar to those seen in RA [4]. One study reported that 18 patients of 22 patients with UC have specific radiological osseous abnormalities [11]. The computed tomography (CT) shows better sensitivity than x-ray analysis and can detect the changes of early sacroiliitis before they became apparent on plain films: bilateral, symmetrical joint narrowing with osseous erosion and then sclerosis [12,13].

As previously mentioned, the arthritis is pauciarticular. Arthritis begins in the lower limb and the most frequently affected joints are knees, ankles, hips, and elbows, while the finger-joints are less commonly attacked [6,14-16].

There is a positive association between arthritis and UC in the duration and extent of symptoms as arthritis usually subsides with the remission of UC, especially after a colectomy and exacerbations of arthritis [3,6,17].

In another three studies, each one gave a case report with a long history of RA, which was followed by the association of UC, based on endoscopic and histological findings. However, the underlying mechanism was unknown [18-20].

Genetics and Antigens

The relationship between UC and RA has not yet become clear. Perhaps specific genes may predispose to both diseases; however; no genetic risk factor has been identified [21].

Some studies suggested that UC and RA are both associated with the human leukocyte antigen (HLA-B27) [22]. Klausen et al. reported a patient with both UC and RA who had a positive association with HLA-B27 [23]. Scarpa et al. studied 79 patients with UC and found a higher prevalence of HLA-B27 (p <0.05) [24]. Núñez et al. indicated that the development of articular manifestations in patients with ulcerative colitis was influenced by genetic factors in the major histocompatibility complex haplotypes [25]. However, while Scarpa et al. and Núñez et al. make the same points, the latter is better as it contains a greater number of patients and is more recent [24,25].

In a 2001 study, Mosquera-Marinez et al. state that interleukin 15 is overexpressed in the inflamed mucosa of patients with inflammatory bowel disease at the level of macrophages. Also, the study reported that interleukin 15 was present in patients with RA [26].

In 1988, Cooper et al. performed a study to measure the serum titers of IgA in many inflammatory diseases, revealing that the levels are increased in RA and UC [27].

Chen at al. in their 2008 study “Haplotypes of PADI4 susceptible to RA are also associated with UC in the Japanese population” studied the haplotypes of peptidyl arginine deiminase type 4 (PADI4) in 114 patients with UC. The results showed that haplotype 2 of (PADI4) is susceptible to UC. Also, the study indicated that the haplotypes of PADI4 are the RA-susceptible gene [28].

Perdigones performed a study in 2010, on the regulatory element of the prostaglandin receptor ER4 on 662 UC patients and 605 RA patients. The prostaglandin receptor ER4 is associated with UC. The result of this study discovered that there is a significant influence between these polymorphisms and UC and RA predisposition [29].

Effect of Certain Drugs on Ulcerative Colitis and Rheumatoid Arthritis

Many drugs are used for the treatment of both UC and RA. One of these drugs is corticosteroid, which reduces colonic inflammation and reduces the incidence of RA [9,30].

Asada et al. discussed the effect of the therapeutic agents of RA on patients with UC [19]. Some of these drugs did not induce colitis, although gold salts induced a form of colitis resembling UC. At the end of this study, they reported that the therapeutic medications for RA are unlikely to be the underlying cause of colitis [19].

Ruggiero et al.’s 2009 study reported that the omega-3 polyunsaturated fatty acid (PUFA) in large doses affects patients with UC and RA. Omega-3 (PUFA) is beneficial in inflammatory diseases by reducing pain, the number of tender joints, and the duration of stiffness [31].

In 2016, Szeto et al. showed that tocilizumab is an interleukin-6 receptor inhibitor for moderate and severe rheumatoid arthritis, and they used it for patients with RA and UC. They found clinical improvement in both diseases, and the laboratory studies supported the role of interleukin-6 in the pathophysiology of ulcerative colitis [32].

So, we conclude that there is a group of drugs which can be used in the treatment of either UC or RA. Some of these drugs are used as therapeutic agents for patients who simultaneously have both diseases, and this proves that there is a link or relation between UC and RA.

Coexistence of Rheumatoid Arthritis and Ulcerative Colitis

We studied 15 studies which included data on the number of patients with both UC and RA and collated the data in Table 1 below.

**Table 1:** The number of patients with ulcerative colitis and rheumatoid arthritis

Study	Number of patients with ulcerative colitis	Number of patients with rheumatoid arthritis	Notes
Ansell et al. [33]	91 (37 males +54 females)	18 (7 males +11 females)	
Wright et al. [6]	269	31	50% rheumatic complain
Russell et al. [34]	42	4	With x-ray evidence
Nugent et al. [35]	555	17% (for all types of arthritis)	All the 17% have articular manifestations respectively rheumatoid arthritis, rheumatoid spondylitis, arthralgia, erythema nodosum
Lindsley et al. [36]	86	18 (21%)	
Passo et al. [14]	44	4	
Mosebach et al. [37]	30	21%	
Bernstein et al. [38]	3873	30.9%	

Two studies assessed the risk of RA in patients with UC. The first study (Weng et al.) said that the risk of RA was 1.9 (95% CI: 1.5-2.3) but the second study (Cohen et al.) said that it was 2.1 (1.8-2.3) [39,40]. The first study seems more accurate as it included more patients (12,601) and was performed over a longer period (1996-2005); the second study was conducted from 2001 to 2002.

Yüksel et al. enrolled 357 patients with inflammatory bowel disease (IBD) in their research and showed that 66 patients (18.5%) had IBD-related peripheral arthritis (13.5% ulcerative colitis) and the most common places were the knee (65.2%) and ankle (62.1%) [41].

In the study by Zippi et al., 595 patients with UC were enrolled, and the results showed that 168 patients had musculoskeletal manifestations, which included 61 with arthritis type 1 and 100 with arthritis type 2 [42].

Finally, two other studies revealed the odd's ratio of the presence of RA with UC. Wilson et al. reported an odd's ratio of 1.9 (95% CI: 1.5-2.3) [43]. Bae et al. included 28,197 patients with UC, collected data from 2009 to 2013, and reported an increased risk of RA (OR: 3.474, 95% CI: 2.671-4.519) [44].

These two studies provided further evidence that there is a relation between UC and RA [45,46]. Each one carried a case report about a patient with UC, and then these cases were complicated by arthritis that resembles that clinical picture of RA [45,46].

Studies Disagreeing on the Relation between Rheumatoid Arthritis and Ulcerative Colitis

On the other side, some studies disagree with the presence of the relationship between the two diseases. Hammer et al. noted that the polyarthritis of patients with UC is different in many aspects from that of RA [47]. McEwen showed that arthritis connected with UC differs from that of RA in being acute in onset, involving few joints and recovering without residual changes [48]. Finally, Teleuolova et al. showed that the combination of UC and RA is rare [49]. However, the number of studies which disagree with this study’s proposal is tiny against the number of studies that prove that there is a relation between the two diseases. 

The limitations of this study are that this study is a traditional review, not a systematic review, and we did not include studies outside English-language. We recommend that future studies will focus on this relationship and discovering how both diseases affect each other. We also recommend that future studies be based on clinical trials or case reports as there is currently only a small number of these studies.

## Conclusions

We aimed to find the relationship between UC and RA and how they affect each other. This review discusses the characteristics of arthritis in patients with UC, which resemble that of RA. We also collected data on the number of patients that have both conditions from many studies. All these findings provide sufficient evidence that there is a relation between the two diseases. This study is beneficial as it reviews data collected from the last 50 years from different databases, including all cases that have both diseases. It will help scientists and doctors to understand individual and shared elements of both disorders better.

## References

[1] Adams SM, Bornemann PH (2013). Ulcerative colitis. Am Fam Physician.

[2] Charles J, Britt H, Pan Y (2013). Rheumatoid arthritis. Aust Fam Physician.

[3] Edwards FC, Truelove SC (1964). The course and prognosis of ulcerative colitis. III. Complications. Gut.

[4] Bywaters EGL, Ansell BM (1958). Arthritis associated with ulcerative colitis; a clinical and pathological study. Ann Rheum Dis.

[5] Colìa R, Corrado A, Cantatore FP (2016). Rheumatologic and extraintestinal manifestations of inflammatory bowel diseases. Ann Med.

[6] Wright V, Watkinson G (1965). The arthritis of ulcerative colitis. Br Med J.

[7] Raffucci FL (1965). Colitis and arthritis. Br Med J.

[8] Momohara S (1999). [Arthritic manifestations in ulcerative colitis] (Article in Japanese). Nihon Rinsho.

[9] Jalan KN, Prescott RJ, Walker RJ, Sircus W, McManus JP, Card WI (1970). Arthropathy, ankylosing spondylitis, and clubbing of fingers in ulcerative colitis. Gut.

[10] Gravallese EM, Kantrowitz FG (1988). Arthritic manifestations of inflammatory bowel disease. Am J Gastroenterol.

[11] Clark RL, Muhletaler CA, Margulies SI (1971). Colitic arthritis. Clinical and radiographic manifestations. Radiology.

[12] Cammisa M, Lomuto M, Bonetti MG (1987). Sacroiliitis in seronegative polyarthritis: CT analysis. Clin Exp Rheumatol.

[13] Gore RM, Balthazar EJ, Ghahremani GG, Miller FH (1996). CT features of ulcerative colitis and Crohn's disease. AJR Am J Roentgenol.

[14] Passo MH, Fitzgerald JF, Brandt KD (1986). Arthritis associated with inflammatory bowel disease in children. Relationship of joint disease to activity and severity of bowel lesion. Dig Dis Sci.

[15] (1958). Arthritis in ulcerative colitis. Br Med J.

[16] Hendrickson BA, Gokhale R, Cho JH (2002). Clinical aspects and pathophysiology of inflammatory bowel disease. Clin Microbiol Rev.

[17] Russell AS (1977). Arthritis, inflammatory bowel disease, and histocompatibility antigens. Ann Intern Med.

[18] Adachi Y, Hinoda Y, Takahashi Takahashi (1996). Rheumatoid arthritis associated with ulcerative colitis. J Gastroenterol.

[19] Asada Y, Isomoto H, Shikuwa S (2006). Development of ulcerative colitis during the course of rheumatoid arthritis: association with selective IgA deficiency. World J Gastroenterol.

[20] Hemminki K, Li X, Sundquist K, Sundquist J (2010). Familial association of inflammatory bowel diseases with other autoimmune and related diseases. Am J Gastroenterol.

[21] Toyoda H, Wang SJ, Yang HY (1993). Distinct associations of HLA class II genes with inflammatory bowel disease. Gastroenterology.

[22] Moll JM (1985). Inflammatory bowel disease. Clin Rheum Dis.

[23] Klausen T, Amris K, Helin P (1992). Ulcerative colitis complicating seronegative HLA-A2-B27 rheumatoid arthritis with sacroiliitis. Ann Rheum Dis.

[24] Scarpa R, del Puente A, D'Arienzo A (1992). The arthritis of ulcerative colitis: clinical and genetic aspects. J Rheumatol.

[25] Núñez C, Alecsandru DM, Mendoza JL, Urcelay E, Díaz-Rubio M, de la Concha EG, Martinez A (2006). Genetic markers linked to rheumatoid arthritis are also strongly associated with articularmanifestations in ulcerative colitis patients. Hum Immunol.

[26] Mosquera-Martinez J, Boyer F, Fontanges E, Miossec P (2001). Rheumatoid arthritis associated with ulcerative colitis. Ann Rheum Dis.

[27] Cooper R, Fraser SM, Sturrock RD, Gemmell CG (1988). Raised titres of anti-klebsiella IgA in ankylosing spondylitis, rheumatoid arthritis, and inflammatory bowel disease. Br Med J (Clin Res Ed).

[28] Chen CC, Isomoto H, Narumi Y (2008). Haplotypes of PADI4 susceptible to rheumatoid arthritis are also associated with ulcerative colitis in the Japanese population. Clin Immunol.

[29] Perdigones N, Martín E, Robledo G (2010). Study of chromosomal region 5p13.1 in Crohn's disease, ulcerative colitis, and rheumatoidarthritis. Hum Immunol.

[30] Burt RW, Berenson MM, Samuelson CO, Cathey WJ (1983). Rheumatoid vasculitis of the colon presenting as pancolitis. Dig Dis Sci.

[31] Ruggiero C, Lattanzio F, Lauretani F, Gasperini B, Andres-Lacueva C, Cherubini A (2009). Omega-3 polyunsaturated fatty acids and immune-mediated diseases: inflammatory boweldisease and rheumatoid arthritis. Curr Pharm Des.

[32] Szeto MCH, Yalçın MD, Khan A, Piotrowicz A (2016). Successful use of tocilizumab in a patient with coexisting rheumatoid arthritis and ulcerative colitis. Case Reports Immunol.

[33] Ansell BM, Wigley RAD (1964). Arthritic manifestations in regional enteritis. Ann Rheum Dis.

[34] Russell AS (1965). Arthritis and ulcerative colitis. Br Med J.

[35] Nugent FW, Rudolph NE (1966). Extracolonic manifestations of chronic ulcerative colitis. Med Clin North Am.

[36] Lindsley CB, Schaller JG (1974). Arthritis associated with inflammatory bowel disease in children. J Pediatr.

[37] Mosebach S, Tromm A, Wittenborg A, May B (1995). [Rheumatoid disorders in Crohn disease and ulcerative colitis. Dominance of non-inflammatoryfactors] (Article in German). Leber Magen Darm.

[38] Bernstein CN, Wajda A, Blanchard JF (2005). The clustering of other chronic inflammatory diseases in inflammatory bowel disease: a population-based study. Gastroenterology.

[39] Weng X, Liu L, Barcellos LF, Allison JE, Herrinton LJ (2007). Clustering of inflammatory bowel disease with immune mediated diseases among members of a northern california-managed care organization. Am J Gastroenterol.

[40] Cohen R, Robinson D Jr, Paramore C, Fraeman K, Renahan K, Bala M (2008). Autoimmune disease concomitance among inflammatory bowel disease patients in the United States, 2001-2002. Inflamm Bowel Dis.

[41] Yüksel I, Ataseven H, Başar O (2011). Peripheral arthritis in the course of inflammatory bowel diseases. Dig Dis Sci.

[42] Zippi M, Corrado C, Pica R (2014). Extraintestinal manifestations in a large series of Italian inflammatory bowel disease patients. World J Gastroenterol.

[43] Wilson JC, Furlano RI, Jick SS, Meier CR (2016). Inflammatory bowel disease and the risk of autoimmune diseases. J Crohns Colitis.

[44] Bae JM, Choo JY, Kim KJ, Park KS (2017). Association of inflammatory bowel disease with ankylosing spondylitis and rheumatoid arthritis: a nationwide population-based study. Mod Rheumatol.

[45] Speiser JC, Moore TL, Zuckner J (1985). Ulcerative colitis with arthritis and vasculitis. Clin Rheumatol.

[46] Israel DM, Olson AD, Ilowite NT, Davidson M (1989). Arthritis as the initial manifestation of inflammatory bowel disease in early infancy. J Pediatr Gastroenterol Nutr.

[47] Hammer B, Ashurst P, Naish J (1968). Diseases associated with ulcerative colitis and Crohn's disease. Gut.

[48] McEwen C (1968). Arthritis accompanying ulcerative colitis. Clin Orthop Relat Res.

[49] Teleuolova AS, Beysenbekova ZhA, Tayzhanova DZh, Teuesheva ZB, Guseinova ZK (2015). [Nonspecific ulcerative colitis in combination with rheumatoid arthritis (case report)] (Article in Russian). Georgian Med News.

